# Association between initial dialytic modalities and the risks of mortality, infection death, and cardiovascular events: A nationwide population-based cohort study

**DOI:** 10.1038/s41598-020-64986-2

**Published:** 2020-05-15

**Authors:** Yi-Ran Tu, Tsung-Yu Tsai, Ming-Shyan Lin, Kun-Hua Tu, Cheng-Chia Lee, Victor Chien-Chia Wu, Hsiang-Hao Hsu, Ming-Yang Chang, Ya-Chung Tian, Chih-Hsiang Chang

**Affiliations:** 1 Kidney Research Center, Department of Nephrology, Chang Gung Memorial Hospital, Taoyuan, Taiwan; 2 Graduate Institute of Clinical Medical Science, College of Medicine, Chang Gung University, Taoyuan, Taiwan; 3 Devision of Cardiology, Department of Internal Medicine, Chang Gung Memorial Hospital, Yulin, Taiwan; 4 Department of Cardiology, Chang Gung Memorial Hospital, Taoyuan, Taiwan

**Keywords:** Haemodialysis, Peritoneal dialysis

## Abstract

To date, few studies have been conducted to pairwise compare the prognosis of peritoneal dialysis (PD), unplanned PD, and unplanned hemodialysis (HD). We analyzed longitudinal data from Taiwan’s National Health Insurance Research Database. We included 45,165 patients whose initial dialytic modality was PD or unplanned HD between January 1, 2001 and December 31, 2013. We divided the patients into three groups according to their initial dialytic modalities. The primary outcomes were all-cause mortality and death from infection during 1-year follow up. The risks of all-cause mortality and infection death were higher in the unplanned PD group than in the planned PD group (hazard ratio [HR] 1.43, 95% confidence interval [CI] 1.28–1.60; HR 1.54, 95% CI 1.32–1.80). Likewise, the risks of all-cause mortality and infection death were higher in the unplanned HD group (HR 1.64, 95% CI 1.48–1.82; HR 1.85, 95% CI 1.61–2.13). Furthermore, the risks of all-cause mortality and infection death were also higher in the unplanned HD group than in the unplanned PD group (HR 1.15, 95% CI 1.07–1.23; HR 1.20, 95% CI 1.09–1.32). In conclusion, our study demonstrates that patients whose initial modality was planned PD or unplanned PD may have better clinical outcomes than those whose initial modality was unplanned HD.

## Introduction

The renal replacement therapy (RRT) includes hemodialysis (HD), peritoneal dialysis (PD), and renal transplantation. Until now, it is still controversial regarding whether HD or PD is the more appropriate dialysis modality with better outcomes. Some studies showed PD had superior outcomes than HD^1,2^. On the other hand, some studies demonstrated better outcomes in HD than in PD^3–5^. Thiery et al. mentioned that planned HD had a better survival rate than planned PD in a 5-year follow-up period. Sim JJ et al. showed that PD had the lowest 6-month mortality risk compared to planned HD^6^. Ben Wong et al. showed that HD and PD had the similar mortality among incident dialysis patients^7^.

End-stage renal disease (ESRD) with urgent need for dialysis is common among late-referred patients, particularly those older. The unplanned initiation of renal replacement therapy remains a major concern for these patients.

In acute unplanned settings, hemodialysis is often preferred at dialysis initiation among incident HD patients^8^. These patients are particularly likely to be started on in-center HD with a temporary catheter; we term such treatment as “unplanned HD.” Patients on unplanned HD are known to be associated with a higher mortality and increased risks of lethal complications, such as bacteremia and central venous thrombosis or stenosis. A study observed that in unplanned dialysis initiation by temporary HD among incident dialysis patients, catheters are independently associated with both greater mortality and a high rate of infectious complications^9^. Infectious complications represent a major cause of morbidity and is the second leading cause of death in dialysis populations^10,11^. Previous study demonstrated that planned HD with usage of arteriovenous fistula (AVF) had a lower risk for death, and unplanned HD with usage of catheter had a higher risk for death compared with patients on PD^12^.

Unlike PD with a planned start, unplanned PD is preceded by temporary HD in order to relieve uremic symptoms. An unplanned start may be associated with a shorter time of peritoneal infusion and slightly increased risk of leakage from an unhealing wound due to a shorter duration between catheter insertion and dialysate infusion.

To date, few studies have compared the outcomes of unplanned HD and unplanned PD. The aim of the study was to explore the prognosis among unplanned HD, unplanned PD, and planned PD.

## Results

### Study population characteristics

Table 1 details the baseline characteristics of the study population, stratified by the initial modalities of planned PD, unplanned PD, and unplanned HD. Specifically, 6,746 patients were receiving planned PD, 8,555 patients were receiving unplanned PD, and 25,762 patients were receiving unplanned HD. Before adjusting for multiple propensity scores, there were large differences in baseline characteristics among the study groups, which became well-balanced after multiple propensity scores were adjusted for, with the exception of age, number of prior nephrologist outpatient visits, heart failure, myocardial infarction, and beta-blocker (*P* < 0.05). The mean follow up duration was 5 years in the planned PD group, 4.3 years in the unplanned PD group and 2.1 years in the planned HD group, respectively.

**Table 1:** Baseline characteristics of patients.

Variable	Planned PD (*n* = 6,746)	Unplanned PD (*n* = 8,555)	Unplanned HD (*n* = 25,762)	*P* value of univariate	*P* value of multivariate#
Demographic					
Age (years)	54.6 ± 14.4	54.2 ± 15.9	67.8 ± 14.0	<0.001	0.035
Age ≥ 65 years	1,679 (24.9)	2,228 (26.0)	15,653 (60.8)	<0.001	0.089
Male	3,044 (45.1)	4,147 (48.5)	12,690 (49.3)	<0.001	0.868
No. of prior nephrologist outpatient visit in the previous year	13.9 ± 9.3	9.7 ± 8.9	8.5 ± 8.6	<0.001	0.001
Monthly income, NTD				<0.001	0.965
0 - 17,880	2,238 (33.2)	2,900 (33.9)	10,444 (40.5)		
17,881 – 22,800	2,104 (31.2)	2,734 (32.0)	9,407 (36.5)		
>22,800	2,404 (35.6)	2,921 (34.1)	5,911 (22.9)		
Comorbidity					
Hypertension	5,486 (81.3)	7,417 (86.7)	23,417 (90.9)	<0.001	0.971
Diabetes mellitus	2,373 (35.2)	3,585 (41.9)	16,869 (65.5)	<0.001	0.890
Chronic obstructive pulmonary disease	278 (4.1)	342 (4.0)	2,717 (10.5)	<0.001	0.114
Peripheral arterial disease	140 (2.1)	216 (2.5)	1,400 (5.4)	<0.001	0.815
Ischemic heart disease	1,058 (15.7)	1,757 (20.5)	8,566 (33.3)	<0.001	0.107
Polycystic kidney disease	170 (2.5)	126 (1.5)	368 (1.4)	<0.001	0.598
History of event					
History of heart failure	774 (11.5)	1,601 (18.7)	9,311 (36.1)	<0.001	0.001
Previous ischemic stroke	550 (8.2)	853 (10.0)	6,194 (24.0)	<0.001	0.306
Previous hemorrhage stroke	95 (1.4)	177 (2.1)	1,042 (4.0)	<0.001	0.655
Old myocardial infarction	198 (2.9)	439 (5.1)	2,854 (11.1)	<0.001	0.035
CCI score	3.5 ± 1.7	3.9 ± 1.9	5.1 ± 2.0	<0.001	0.126
Medications					
Antiplatelet	1,087 (16.1)	1,717 (20.1)	8,588 (33.3)	<0.001	0.435
ACEi/ARB	3,269 (48.5)	4,867 (56.9)	12,445 (48.3)	<0.001	0.158
Beta-blocker	3,597 (53.3)	4,716 (55.1)	12,399 (48.1)	<0.001	0.012
Loop diuretics	3,340 (49.5)	5,186 (60.6)	14,953 (58.0)	<0.001	0.300
Oral hypoglycemic agent	1,358 (20.1)	2,070 (24.2)	9,305 (36.1)	<0.001	0.614
Insulin	1,081 (16.0)	1,496 (17.5)	6,702 (26.0)	<0.001	0.749
Statin	1,966 (29.1)	2,257 (26.4)	5,873 (22.8)	<0.001	0.665
Follow-up duration (years)	5.0 ± 3.7	4.3 ± 3.4	2.1 ± 2.1	<0.001	0.402

PD, peritoneal dialysis; HD, hemodialysis; NTD, national Taiwan dollar; CCI, Charlson comorbidity index; ACEi, angiotensin converting enzyme inhibitor; ARB, angiotensin receptor blocker;

^#^Adjusted for multiple propensity scores;

Data were presented as frequency (percentage) or mean ± standard deviation.

### Clinical outcomes

During the entire follow up, 33.2 (5,074/15,301) of the patients with initial PD switched to HD and 156 (0.61), whereas 0.61% (156/25,762) of the patients with initial HD switched to PD (data not shown).

Table 2 summarizes the pairwise comparisons of 1-year outcomes among the study groups. The risks of all-cause mortality and infection death were higher in the unplanned PD group than in the planned PD group (hazard ratio [HR] 1.43, 95% confidence interval [CI] 1.28–1.60; subdistribution HR [SHR] 1.50, 95% CI 1.29–1.75, respectively). Likewise, the risks of all-cause mortality and infection death were higher in the unplanned HD group than in the planned PD group (HR 1.64, 95% CI 1.48–1.82; SHR 1.82, 95% CI 1.58–2.10, respectively). Furthermore, the risks of all-cause mortality and infection death were also higher in the unplanned HD group than in the unplanned PD group (HR 1.15, 95% CI 1.07–1.23; SHR 1.21, 95% CI 1.10–1.34, respectively).

**Table 2:** Time to event outcome analysis during the 1-year follow up.

Outcome	Number of event (%)			Adjusted HR or SHR (95% CI) #		
	Planned PD (*n* = 6,746)	Unplanned PD (*n* = 8,555)	Unplanned HD (*n* = 25,762)	Unplanned PD vs. Planned PD (reference)	Unplanned HD vs. Planned PD (reference)	Unplanned HD vs. Unplanned PD (reference)
All-cause mortality	475 (7.0)	1,018 (11.9)	6,975 (27.1)	1.43 (1.28–1.60)*	1.64 (1.48–1.82)*	1.15 (1.07–1.23)*
Infection death	241 (3.6)	556 (6.5)	4,215 (16.4)	1.50 (1.29–1.75)*	1.82 (1.58–2.10)*	1.21 (1.10–1.34)*
MACCE§	413 (6.1)	812 (9.5)	5,310 (20.6)	1.29 (1.14–1.46)*	1.61 (1.45–1.80)*	1.25 (1.15–1.36)*
All-cause admission	3,133 (46.4)	4,601 (53.8)	16,079 (62.4)	1.18 (1.13–1.23)*	1.31 (1.25–1.37)*	1.11 (1.07–1.16)*

PD, peritoneal dialysis; HD, hemodialysis; HR, hazard ratio; SHR, subdistribution hazard ratio; MACCE, major adverse cardiac and cerebrovascular event;

^§^Including acute myocardial infarction, acute ischemic stroke, intracerebral hemorrhage, heart failure, or cardiovascular death;

^#^Adjusted for multiple propensity scores, age, number of prior nephrologist outpatient visit in the previous year, dementia, heart failure, myocardial infarction, use of proton pump inhibitor and use of beta-blocker;

^*^P value < 0.05;

Data were presented as frequency (percentage).

Regarding major cardiac and cerebrovascular events (MACCE) and all-cause readmission, the risks were greater in the unplanned PD group than in the planned PD group (HR 1.29, 95% CI 1.14–1.46; SHR 1.18, 95% CI 1.13–1.23, respectively). Likewise, the risks of MACCE and all-cause readmission were greater in the unplanned HD group than in the planned PD group (HR 1.61, 95% CI 1.45–1.80; SHR 1.31, 95% CI 1.25–1.37, respectively). On the other hand, the risks of MACCE and all-cause readmission were also greater in the unplanned HD group than in the unplanned PD group (HR 1.25, 95% CI 1.15–1.36; SHR 1.11, 95% CI 1.07–1.16, respectively) (Table 2).

**Figure 1:**
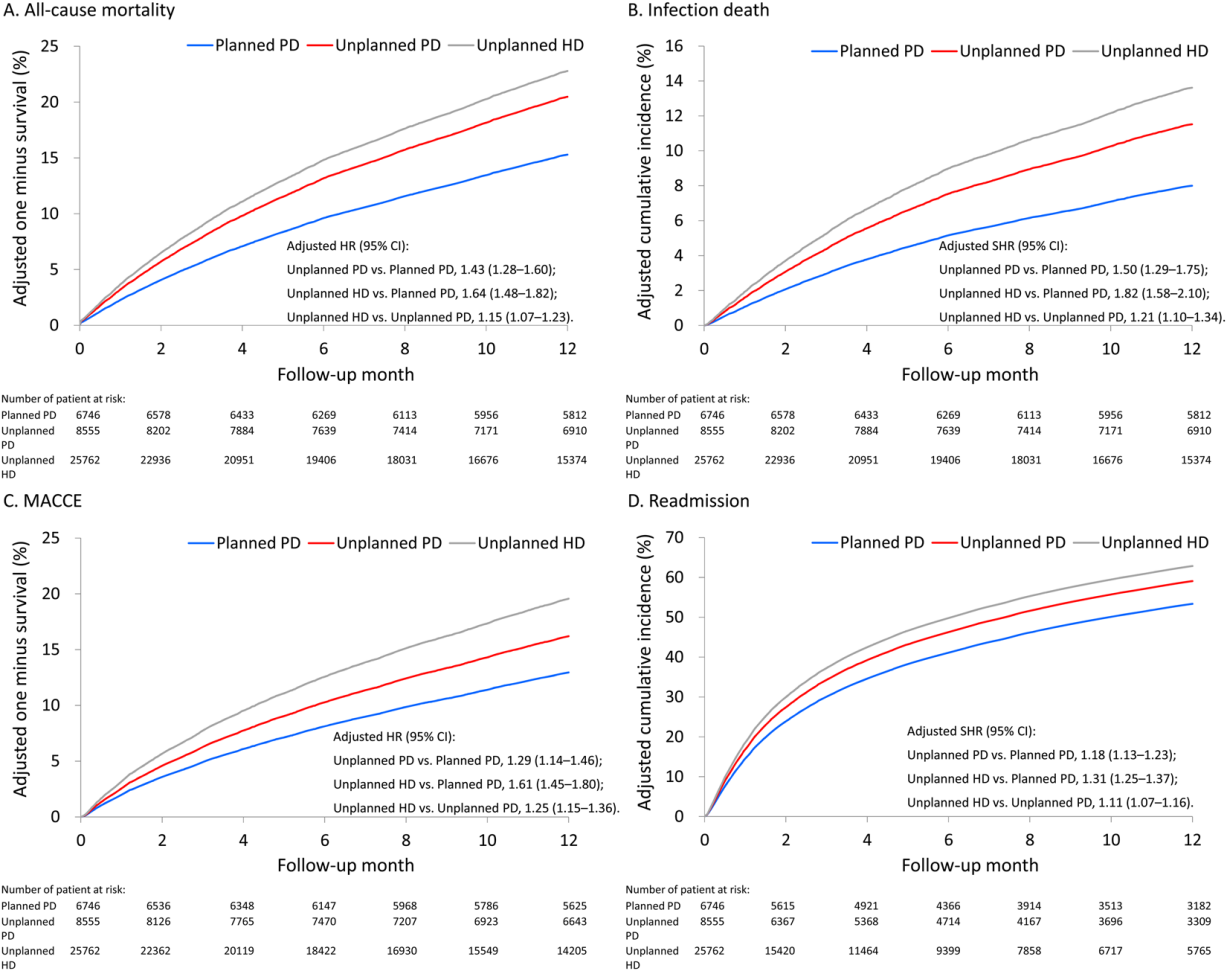
Direct-adjusted (predicted) survival of all-cause mortality (**A**) and major cardiac and cerebrovascular events (**C**), and direct-adjusted cumulative incidence function of infection death (**B**) and all-cause readmission (**D**) among patients with different initial dialytic modalities during the 1-year follow-up.

The direct-adjusted survival or cumulative incidence functions for each dialytic modality and each outcome during the 1-year follow up are depicted in Fig. 1A–D, respectively.

As a supplemental analysis, we compared the risks of clinical outcomes between the planned PD and planned HD groups. In the patients with planned dialysis, the planned HD group was predominant (88.6%; 52,600/59,346). The baseline characteristics were balanced after matching between groups (Supplemental Table 2). Comparing to the planned HD group, the planned PD group had borderline significantly lower risks of 1-year all-cause mortality (HR 0.88, 95% CI 0.78–1.0001). Likewise, the planned PD group had significantly lower risks of MACCE than did the planned HD group (HR 0.80, 95% CI 0.70–0.91). The risks of infection death were comparable between these two groups. However, the risks of all-cause admission were significantly greater in the PD group (SHR 1.14, 95% CI 1.09–1.20) (Supplemental Table 3).

## Discussion

This current study analyzed the outcomes of unplanned HD, unplanned PD and planned PD in a national wide population. The all-cause mortality, death from infection, MACCE, and all-cause admission were lowest in patients with planned PD, followed by unplanned PD and unplanned HD. In addition, comparing to the planned HD group, the planned PD group had lower risks of MACCE, borderline significantly lower risks of 1-year all-cause mortality.

Our study demonstrated the protective effect of avoiding unplanned HD. An observational cohort study by Michael and Matthias et al. focused on incident dialysis patients with either unplanned and acute PD (n = 466) or HD (n = 457) in a single center from March 2005 to June 2010; there was a 6-month follow-up. The study observed that although the dialysis modality in an acute unplanned dialysis setting had no significant influence on survival, patients with HD had a significantly higher risk of bacteremia, perhaps due to the use of a central venous dialysis catheter. Thus, the authors suggested that PD is a safe and efficient alternative to HD in acute unplanned dialysis settings^8^. Although for Michael and Matthias et al., acute unplanned PD was designed to reduce the use of a tunneled catheter—thus differing from our definition—our study argued that in acute unplanned settings, PD remains better than HD.

Another term in the literature is “urgent start PD,” which refers to the use of PD catheters within 48–72 hours after insertion, in combination with various protocols to reduce complication. Its more elective variant, where PD is initiated between 3 and 14 days after catheter insertion, is best termed “early start PD,” and it is predominantly an outpatient and less stressful procedure^13^. “Urgent start PD” or “early start PD” do not equal to “unplanned PD”. However, the urgent start concept still also can be used among unplanned PD patients to reduce temporary HD use.

There are some possible explanations why unplanned HD has the most unfavorable outcomes compared to either planned or unplanned PD. First, the use of a tunneled cuffed catheter (TCC) in unplanned HD is associated with chronic inflammation (whether with or without clinical infection), which can lead to atherosclerosis progression. According to several studies, such as Raad^14^ and Jones^15^, the colonization of all TCCs, with no evidence of clinical infection, by bacteremia and biofilm accompany the long-term use of a central vein catheter (CVC). According to Coli et al., unlike AVF or arteriovenous graft, colonization by an organism, and possibly the TCC itself, may lead to chronic inflammation^16^. Moreover, as demonstrated by Libby^17^, chronic inflammation status is an important risk factor for endothelial dysfunction and its subsequent atherosclerosis progression.

Second, according to both a United States Renal Data System (USRDS) Wave 2 study^18^ and an older study by Powe^19^, the catheter used in temporary and permanent HD is associated with a higher risk of bacteremia when compared with the risk from PD or planned HD. In the same study, Ishani et al. also demonstrated that bacteremia itself has an adverse effect with regard to patient survival, myocardial infarction, heart failure, and peripheral vascular disease^18^.

Third, among the different catheters used in dialysis, TCC has a higher risk of dysfunction, which may lead to higher risks of mortality and cardiovascular (CV) events. According to two studies^20,21^, access type and complication (whether infection or non-infection) resulted in higher patient mortality and occurrences of CV events. Kou also revealed that, after comorbidities were adjusted for, patients with access dysfunction had a higher odds ratio (of 1.268) for major adverse CV events^22^.

Fourth, Liebman et al. suggested that some (11%) patients started HD with a long-term use of a CVC due to immature AVF^23^. We considered these selected patients of Liebman’s study who use CVC as HD assess to share the same risk factor for the occurrence of a CV event, mortality, and immature AVF (included hypertension, diabetes mellitus, and CV disease)^24,25^. This potentially explains the higher risks of a CV event and death from unplanned HD that were observed in the current study. The current study also demonstrated a diminished protective effect of PD and unplanned HD after 5 years. To explain this finding, some patients shift to HD because of either technical failure or a fear of the possibility of encapsulating peritoneal sclerosis from long-term PD.

Our study also found that comparing to the planned HD group, the planned PD group had lower risks of MACCE, borderline significantly lower risks of 1-year all-cause mortality. However, the planned PD group had higher rate of first-year readmission compared to the planned HD group. Previously, Jeffery et al. showed the risk for 30-day readmission is higher for patients with PD compared to in-center HD therapy. The authors suggested that the higher readmission rate in PD relative to in-center HD may be due to patients receiving in-center HD having earlier and frequent medical care several times per week but much less frequent medical care for patients with home-based PD, typically once per month^12^.

Our study has several limitations. First, causality cannot be inferred due to the retrospective nature of this study’s use of the Taiwan’s National Health Insurance (NHI) Research Database (NHIRD). Second, we had no access to personal data, such as those on family medical history and a patient’s lifestyle (e.g., whether a patient smokes), and laboratory data, such as blood pressure control or lipid data. Such data would have allowed us to account for these risk factors of mortality and CV events. Third, we did not know the exact cause of death in instances of death from infection (such as death due to pneumonia or catheter-related infection), disallowing inference on its relationship to dialysis modality. Fourth, the modality shift between PD and HD was not evaluated, which might interfere with the outcome of our study.

## Conclusion

Patients with advanced chronic kidney disease who were receiving PD, whether planned or unplanned, had better clinical outcomes in all-cause mortality, infection death, MACCE, and all-cause admission, than those receiving unplanned HD. Moreover, comparing planned and unplanned PD, planned PD has better clinical outcomes than unplanned PD in all-cause mortality, death from infection, MACCE, and all-cause admission. Although the time event outcomes differed, the first year clinical outcomes were all within the positive hazard ratios (from high to low) of the planned PD, unplanned PD, and unplanned HD.

## Materials and Methods

### Data source

We analyzed longitudinal data from Taiwan’s NHIRD, a dataset containing anonymized health care information that was collected prospectively from 99.9% of Taiwan’s population. Our dataset included patients who started permanent dialysis between 2001 and 2013 in Taiwan. In Taiwan, patients who have started permanent dialysis can receive a catastrophic illness certificate (CIC) that is verified by the Bureau of National Health Insurance, which exempts them from copayments pertaining to their condition. This study was conducted in accordance with the ethical principles of the Declaration of Helsinki. The need for individual consent was waived because personal identification data is not included in the NHIRD. The Institutional Review Board (IRB) of Chang Gung Memorial Hospital approved the study (IRB No. 201901143B1) and also waived the need for informed consent.

### Study population

**Figure 2:**
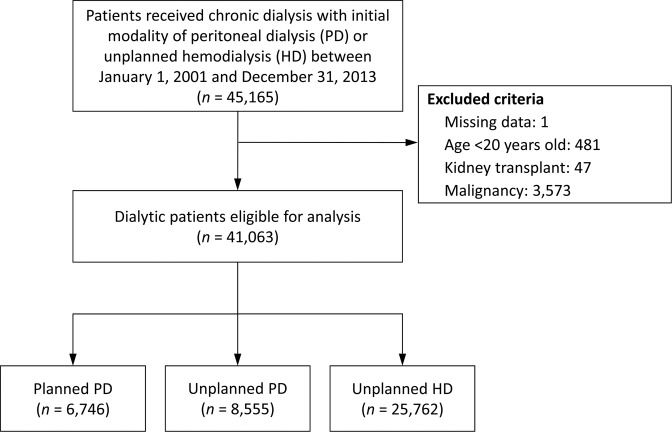
Patient inclusion criteria.

The study population selection process is illustrated in Fig. 2. We included 45,165 patients whose initial dialytic modality (between January 1, 2001 and December 31, 2013) was PD or unplanned HD. We excluded patients who were aged <20 years (n = 481), had missing data (n = 1), and had a history of either renal transplantation (n = 47) or malignancy (n = 3,573). Finally, an eligible study population of 41,063 patients with dialysis were divided into three groups: “planned PD” (n = 6,746), “unplanned PD” (n = 8,555), and “unplanned HD” (n = 25,762). Unplanned PD means that PD initiation was preceded by temporary HD via a catheter in order to relieve uremic symptoms, treat fluid overload, or correct metabolic acidosis and hyperkalemia. By contrast, unplanned HD means that first HD was received via a TCC. For each patient, we determined the modalities based on their Taiwan National Health Insurance reimbursement codes in either the inpatient or outpatient claims database. The date of approval of a patient’s CIC card was defined as the index date.

### Outcome and covariate assessment

We examined the covariates of age, sex, number of nephrologist outpatient visits in the previous year, monthly income, presence of six comorbidities, four events of history, the Charlson Comorbidity Index score, and the use of seven types of medications. Comorbidities were defined in terms of having at least two outpatient diagnoses or any one inpatient diagnosis in the prior year, including hypertension, diabetes mellitus, chronic obstructive pulmonary disease, peripheral artery disease, ischemic heart disease, and dementia. Events of history were present if patients had one inpatient visit before the index date but no earlier than 1997 for conditions including heart failure, ischemic stroke, hemorrhage stroke, and myocardial infarction. Most diagnostic codes used for these diseases have been validated in previous NHIRD-based studies^26–28^. Medications were identified by the filling of its prescription at least once or a refilling of its prescription for a chronic illness at least once within 3 months before or after the index date.

The primary outcomes of interest were all-cause mortality and death from infection. All-cause mortality was also defined as a withdrawal from the NHI program. The cause of death was determined using the inpatient diagnoses or diagnoses from an emergency room, 7 days before the date of withdrawal^29^. The secondary outcomes were MACCE, comprising acute myocardial infarction, acute ischemic stroke, intracerebral hemorrhage, heart failure, and CV death, most diagnostic codes of which have been validated previously^30–34^. The definition of CV death is based on the criteria in the Standardized Definitions for Cardiovascular and Stroke Endpoint Events in Clinical Trials by the United States Food and Drug Administration. MACCE occurrence was defined as the principal diagnosis of hospitalization. In addition, we investigated the first all-cause admission during follow up as another secondary outcome. Each patient was followed from the index date to either the date of event occurrence, date of death, or December 31, 2013, whichever came first.

### Statistical analysis

The baseline characteristics of patients with different dialytic modalities were compared by one-way analysis of variance for continuous variables and chi-square test for categorical variables (the univariate analysis). Because there were substantial differences in the baseline characteristics of the three groups, estimates would be greatly biased if confounders were not adjusted for. We did not choose propensity score matching in this study mainly due to it was not feasible to simultaneously match three groups given the substantial difference of baseline characteristics among groups. It was also not preferable to conduct three pairwise propensity score matching because it would induce the type I error inflation. Furthermore, the matching approach would reduce the generalizability of the results since not all patients were retained in the analysis after matching.

Instead of traditional multivariable adjustment, we conducted an adjustment using multiple propensity scores. First, we constructed a multivariable multinomial logistic model by treating the dialytic modalities as outcome variables and all baseline characteristics (not including the clinical outcomes of interest) as covariates. This model was then used to generate three predicted probabilities (also the propensity scores) for each individual with regard to membership in a given group; these predicted probabilities were then taken as propensity scores. Therefore, group differences related to baseline characteristics could be made small if any two of the three propensity scores were adjusted in the time to event outcome analysis^35^. Compared with traditional multivariable adjustment, adjustment with multiple propensity scores avoids the problem of overfitting, particularly in datasets with few events. As a result, the area under the curve was 85%, 75% and 84% for being in the planned PD, unplanned PD and unplanned HD groups respectively. In addition, the approach of propensity scores adjustment was marginal model whereas the matching approach was conditional model.

To analyze the balance of baseline characteristics among the study groups, after adjustment with multiple propensity scores, a series of multinomial logistic models were applied by treating the dialytic modalities as the outcome variables and each of the baseline characteristics as a covariate (the multivariate analysis). Our observation of insignificance (*P* > 0.05) suggested that there was a well-balance of baseline characteristics among the study groups after adjustment^35^. However, because there is a likelihood of non-balance among the study groups (defined as *P* < 0.10), those variables that have non-balance were further adjusted in the time to event outcome analysis. The risks of mortality and MACCE among the groups were compared with a Cox proportional hazard model. The risk of all-cause readmission and infection death among groups was compared with a Fine and Gray subdistribution hazard model which considered death (for infection death: other causes of death) during the follow up as a competing risk. The survival analyses were additionally adjusted for multiple propensity scores, age, number of prior nephrologist outpatient visits in the previous year, dementia, heart failure, myocardial infarction, use of a proton pump inhibitor, and use of a beta-blocker.

At last, as a supplemental analysis, we compared the risks of clinical outcomes between the planned PD and planned HD group using the propensity score matched cohort with 1:1 matching ratio. The matching was processed using a greedy nearest neighbor algorithm with a caliper of 0.2 times the standard deviation of the logit of the propensity score, with random matching order and without replacement. The balance of covariates between the groups checked using the absolute value of standardized difference (STD) between the groups where a value less than 0.1 is considered negligible difference. In the survival analyses, the within-pair clustering of outcomes after matching was accounted for by using a robust standard error.

A *P* of <0.05 was considered statistically significant, and no adjustment for multiple testing (multiplicity) was made. All statistical analyses were performed using SAS (version 9.4; SAS Institute, Cary, NC), including the “*psmatch*” for propensity score matching and the “*phreg*” package for survival analysis. The direct-adjusted (predicted) survival was derived from the multivariable Cox model with the macro *ADJSURV%*^29^. The direct-adjusted (predicted) cumulative incidence function was obtained from the Fine and Gray model with the macro *CIFCOX%*^36,37^.

## Supplementary information

Supplemental Table 1.

Supplemental Table 2.

Supplemental Table 3.
