# Supplementary material for: Association between initial dialytic modalities and the risks of mortality, infection death, and cardiovascular events: A nationwide population-based cohort study
Source: Sci Rep. 2020 May 15;10:8066. doi: 10.1038/s41598-020-64986-2 (PMC7229162; doi:10.1038/s41598-020-64986-2)
Supplement: Supplementary file 1 — Supplemental Table 1. [file 41598_2020_64986_MOESM1_ESM.docx]

**Association between initial dialytic modalities and the risks of mortality, infection death, and cardiovascular events: A nationwide population-based cohort study**

Yi-Ran Tu^1^, Tsung-Yu Tsai^1,2^, Ming-Shyan Lin^3^, Kun-Hua Tu^1,2^, Cheng-Chia Lee^1,2^, Victor Chien-Chia Wu^4^, Hsiang-Hao Hsu^1^, Ming-Yang Chang^1^, Ya-Chung Tian^1^, Chih-Hsiang Chang^1,2*^

^1^Kidney Research Center, Department of Nephrology, Chang Gung Memorial Hospital, Taoyuan, Taiwan

^2^Graduate Institute of Clinical Medical Science, College of Medicine, Chang Gung University, Taoyuan, Taiwan

^3^Devision of Cardiology, Department of Internal Medicine, Chang Gung Memorial Hospital, Yulin, Taiwan

^4^Department of Cardiology, Chang Gung Memorial Hospital, Taoyuan, Taiwan

* The corresponding author

**Supplemental Table 1**. Time to event outcome analysis during the >1 to 3 year follow up after excluding those who occurred within 1-year follow up or whose follow up duration less than 1 year

|  | Number of event (%) | | |  | HR or SHR (95% CI) | | | | |
| --- | --- | --- | --- | --- | --- | --- | --- | --- | --- |
| Outcome | Planned PD | Unplanned PD | Unplanned HD |  | Unplanned PD  vs.  Planned PD  (reference) |  | Unplanned HD  vs.  Planned PD  (reference) |  | Unplanned HD  vs.  Unplanned PD  (reference) |
| All-cause mortality | 845 (14.6) | 1,017 (14.8) | 3,818 (25.0) |  | 1.04 (0.95–1.14) |  | 2.10 (1.95–2.27)* |  | 2.02 (1.89–2.17)* |
| Infection death | 446 (7.7) | 502 (7.3) | 1,929 (12.6) |  | 0.97 (0.86–1.11) |  | 2.00 (1.80–2.22)* |  | 2.06 (1.86–2.27)* |
| MACCE§ | 615 (11.0) | 753 (11.4) | 2,668 (18.9) |  | 1.06 (0.96–1.18) |  | 2.14 (1.96–2.34)* |  | 2.01 (1.85–2.18)* |
| All-cause admission | 4,226 (75.3) | 5,045 (76.3) | 11,083 (78.5) |  | 0.92 (0.86–0.98)* |  | 0.92 (0.86–0.97)* |  | 1.00 (0.94–1.06) |

HR, hazard ratio; SHR, subdistribution hazard ratio; PD, peritoneal dialysis; HD, hemodialysis; MACCE, major adverse cardiac and cerebrovascular event;

§ Including acute myocardial infarction, acute ischemic stroke, intracerebral hemorrhage, heart failure, or cardiovascular death;

* *P* value <0.05.
