# Supplementary material for: Association between initial dialytic modalities and the risks of mortality, infection death, and cardiovascular events: A nationwide population-based cohort study
Source: Sci Rep. 2020 May 15;10:8066. doi: 10.1038/s41598-020-64986-2 (PMC7229162; doi:10.1038/s41598-020-64986-2)
Supplement: Supplementary file 2 — Supplemental Table 2. [file 41598_2020_64986_MOESM2_ESM.docx]

**Association between initial dialytic modalities and the risks of mortality, infection death, and cardiovascular events: A nationwide population-based cohort study**

Yi-Ran Tu^1^, Tsung-Yu Tsai^1,2^, Ming-Shyan Lin^3^, Kun-Hua Tu^1,2^, Cheng-Chia Lee^1,2^, Victor Chien-Chia Wu^4^, Hsiang-Hao Hsu^1^, Ming-Yang Chang^1^, Ya-Chung Tian^1^, Chih-Hsiang Chang^1,2*^

^1^Kidney Research Center, Department of Nephrology, Chang Gung Memorial Hospital, Taoyuan, Taiwan

^2^Graduate Institute of Clinical Medical Science, College of Medicine, Chang Gung University, Taoyuan, Taiwan

^3^Devision of Cardiology, Department of Internal Medicine, Chang Gung Memorial Hospital, Yulin, Taiwan

^4^Department of Cardiology, Chang Gung Memorial Hospital, Taoyuan, Taiwan

* The corresponding author

**Supplemental Table 2**. Baseline characteristics of patients who received planned PD versus planned HD

|  | Before matching | | |  | After matching | | |
| --- | --- | --- | --- | --- | --- | --- | --- |
| Variable | Planned PD  (*n* = 6,746) | Planned HD  (*n* = 52,600) | STD |  | Planned PD  (*n* = 6,697) | Planned HD  (*n* = 6,697) | STD |
| Demographic |  |  |  |  |  |  |  |
| Age (years) | 54.6 ± 14.4 | 62.3 ± 13.1 | -0.56 |  | 54.7 ± 14.4 | 55.1 ± 14.0 | -0.03 |
| Age ≥ 65 years | 1,679 (24.9) | 23,905 (45.4) | -0.44 |  | 1,679 (25.1) | 1,721 (25.7) | -0.01 |
| Male | 3,044 (45.1) | 25,897 (49.2) | -0.08 |  | 3,023 (45.1) | 3,045 (45.5) | -0.01 |
| No. of prior nephrologist outpatient visit in the previous year | 13.9 ± 9.3 | 10.4 ± 9.3 | 0.38 |  | 13.8 ± 9.3 | 14.1 ± 10.6 | -0.03 |
| Monthly income, NTD |  |  |  |  |  |  |  |
| 0 - 17,880 | 2,238 (33.2) | 19,223 (36.5) | -0.07 |  | 2,223 (33.2) | 2,220 (33.1) | <0.01 |
| 17,881 – 22,800 | 2,104 (31.2) | 20,147 (38.3) | -0.15 |  | 2,094 (31.3) | 2,017 (30.1) | 0.02 |
| > 22,800 | 2,404 (35.6) | 13,230 (25.2) | 0.23 |  | 2,380 (35.5) | 2,460 (36.7) | -0.02 |
| Comorbidity |  |  |  |  |  |  |  |
| Hypertension | 5,486 (81.3) | 41,414 (78.7) | 0.06 |  | 5,441 (81.2) | 5,478 (81.8) | -0.01 |
| Diabetes mellitus | 2,373 (35.2) | 26,066 (49.6) | -0.29 |  | 2,372 (35.4) | 2,464 (36.8) | -0.03 |
| Chronic obstructive pulmonary disease | 278 (4.1) | 3,590 (6.8) | -0.12 |  | 278 (4.2) | 255 (3.8) | 0.02 |
| Peripheral arterial disease | 140 (2.1) | 1,562 (3.0) | -0.06 |  | 140 (2.1) | 140 (2.1) | <0.01 |
| Ischemic heart disease | 1,058 (15.7) | 12,683 (24.1) | -0.21 |  | 1,057 (15.8) | 1,041 (15.5) | 0.01 |
| Polycystic kidney disease | 170 (2.5) | 1,229 (2.3) | 0.01 |  | 170 (2.5) | 183 (2.7) | -0.01 |
| History of event |  |  |  |  |  |  |  |
| History of heart failure | 774 (11.5) | 11,038 (21.0) | -0.26 |  | 773 (11.5) | 777 (11.6) | <0.01 |
| Previous ischemic stroke | 550 (8.2) | 7,181 (13.7) | -0.18 |  | 550 (8.2) | 556 (8.3) | <0.01 |
| Previous hemorrhage stroke | 95 (1.4) | 1,047 (2.0) | -0.05 |  | 95 (1.4) | 93 (1.4) | <0.01 |
| Old myocardial infarction | 198 (2.9) | 2,469 (4.7) | -0.09 |  | 198 (3.0) | 209 (3.1) | -0.01 |
| CCI score | 3.5 ± 1.7 | 4.2 ± 1.9 | -0.36 |  | 3.6 ± 1.7 | 3.6 ± 1.7 | -0.02 |
| Medications |  |  |  |  |  |  |  |
| Antiplatelet | 1,087 (16.1) | 12,602 (24.0) | -0.20 |  | 1,086 (16.2) | 1,115 (16.6) | -0.01 |
| ACEi / ARB | 3,269 (48.5) | 20,113 (38.2) | 0.21 |  | 3,230 (48.2) | 3,241 (48.4) | <0.01 |
| Beta-blocker | 3,597 (53.3) | 21,688 (41.2) | 0.24 |  | 3,557 (53.1) | 3,308 (49.4) | 0.07 |
| Loop diuretics | 3,340 (49.5) | 24,774 (47.1) | 0.05 |  | 3,313 (49.5) | 3,341 (49.9) | -0.01 |
| Oral hypoglycemic agent | 1,358 (20.1) | 14,021 (26.7) | -0.15 |  | 1,358 (20.3) | 1,436 (21.4) | -0.03 |
| Insulin | 1,081 (16.0) | 8,951 (17.0) | -0.03 |  | 1,080 (16.1) | 1,095 (16.4) | -0.01 |
| Statin | 1,966 (29.1) | 9,410 (17.9) | 0.27 |  | 1,924 (28.7) | 1,973 (29.5) | -0.02 |
| Follow-up duration (years) | 5.0 ± 3.7 | 4.9 ± 3.6 | 0.03 |  | 5.0 ± 3.7 | 4.8 ± 3.6 | 0.04 |

PD, peritoneal dialysis; HD, hemodialysis; STD, standardized difference; NTD, national Taiwan dollar; CCI, Charlson comorbidity index; ACEi, angiotensin converting enzyme inhibitor; ARB, angiotensin receptor blocker;

Data were presented as frequency (percentage) or mean ± standard deviation.
