# Supplementary material for: Association between initial dialytic modalities and the risks of mortality, infection death, and cardiovascular events: A nationwide population-based cohort study
Source: Sci Rep. 2020 May 15;10:8066. doi: 10.1038/s41598-020-64986-2 (PMC7229162; doi:10.1038/s41598-020-64986-2)
Supplement: Supplementary file 3 — Supplemental Table 3. [file 41598_2020_64986_MOESM3_ESM.docx]

**Association between initial dialytic modalities and the risks of mortality, infection death, and cardiovascular events: A nationwide population-based cohort study**

Yi-Ran Tu^1^, Tsung-Yu Tsai^1,2^, Ming-Shyan Lin^3^, Kun-Hua Tu^1,2^, Cheng-Chia Lee^1,2^, Victor Chien-Chia Wu^4^, Hsiang-Hao Hsu^1^, Ming-Yang Chang^1^, Ya-Chung Tian^1^, Chih-Hsiang Chang^1,2*^

^1^Kidney Research Center, Department of Nephrology, Chang Gung Memorial Hospital, Taoyuan, Taiwan

^2^Graduate Institute of Clinical Medical Science, College of Medicine, Chang Gung University, Taoyuan, Taiwan

^3^Devision of Cardiology, Department of Internal Medicine, Chang Gung Memorial Hospital, Yulin, Taiwan

^4^Department of Cardiology, Chang Gung Memorial Hospital, Taoyuan, Taiwan

* The corresponding author

**Supplemental Table 3.** Time to event outcome analysis during the 1-year follow up

| Outcome | Planned PD  (*n* = 6,697) | Planned HD  (*n* = 6,697) | HR or SHR of Planned PD  (95% CI) | *P* |
| --- | --- | --- | --- | --- |
| All-cause mortality | 475 (7.1) | 530 (7.9) | 0.88 (0.78–1.0001) | 0.0501 |
| Infection death | 241 (3.6) | 218 (3.3) | 1.10 (0.92–1.32) | 0.313 |
| MACCE§ | 412 (6.2) | 509 (7.6) | 0.80 (0.70–0.91) | 0.001 |
| All-cause admission | 3,111 (46.5) | 2,756 (41.2) | 1.14 (1.09–1.20) | <0.001 |

PD, peritoneal dialysis; HD, hemodialysis; HR, hazard ratio; SHR, subdistribution hazard ratio; MACCE, major adverse cardiac and cerebrovascular event;

§ Including acute myocardial infarction, acute ischemic stroke, intracerebral hemorrhage, heart failure, or cardiovascular death;

Data were presented as frequency (percentage).
